# Supplementary material for: Experimental evidence that thermal selection shapes mitochondrial genome evolution
Source: Sci Rep. 2018 Jun 22;8:9500. doi: 10.1038/s41598-018-27805-3 (PMC6015072; doi:10.1038/s41598-018-27805-3)
Supplement: Supplementary file 1 — Supplementary Information [file 41598_2018_27805_MOESM1_ESM.pdf]

SCIENTIFIC REPORTS: Supplementary Information

Experimental evidence that thermal selection shapes mitochondrial genome evolution

Zdeněk Lajbner [1]*, Reuven Pnini [1], M. Florencia Camus [2,3], Jonathan Miller [1], Damian K. Dowling [2]

[1] Physics and Biology Unit, Okinawa Institute of Science and Technology Graduate University (OIST), 1919-1 Tancha, Onna-son, Okinawa 904-0945, Japan,

[2] School of Biological Sciences, Monash University, Clayton, Victoria 3800, Australia,

[3] Department of Genetics, Evolution & Environment, University College London, London WC1E 6BT, UK.

*Correspondence: Zdeněk Lajbner, Physics and Biology Unit, Okinawa Institute of Science and Technology Graduate University (OIST), 1919-1 Tancha, Onna-son, Okinawa 904-0945, Japan. Phone: +81-(0)98-966-8684, Fax: +81-(0)98-966-1063, Email: z.lajbner@seznam.cz

Emails: reuven.pnini@oist.jp, f.camus@ucl.ac.uk, jm@oist.jp, damian.dowling@monash.edu

Contents

Appendix S1: Detailed methods 2

Supplementary Table S1: Fly food composition 7

Supplementary Table S2: List of samples 8

Supplementary Table S3: Foundation dates of experimental populations 11

Supplementary Table S4: Propagation of experimental populations in dates 13

Supplementary Table S5: Selected SNPs characteristics for mtDNA haplogroups 14

Appendix S1: Detailed methods

Calculations were executed in MATLAB R2014a (MathWorks, Inc., Natick, USA).

Let f_0 be the initial frequency of B mtDNA haplogroup, and f_k $(k = 1,2,\dots,K)$ its frequencies after k generations. The frequencies of two successive generations of selection are related by [45]: $f_{k+1} = f_k(1+s)/(1+sf_k)$ (1)

where s is the selection coefficient. In the case of ATB, we have $f_0 = 0.531 \pm 0.016$ and $f_{fin} = f_0 + \theta_i$ where θ_i $(i = 1,2,3,4)$ is the frequency difference for the four thermal regimes (19°C, 25°C, fluctuating-cold, fluctuating-warm) respectively. Iterating equation (1) according to the number of generations (K=3 generations for the colder regimes, and K=7 for the warmer ones) and solving for s in terms of the initial and final frequencies, we find the results shown in Table A1 (Figs 4 and A1).

In order to evaluate a role of genetic drift in our experiment, we need to estimate female effective population sizes (Nef). Population census size (Nc) did not fluctuate across generations in our experiment. We kept the constant population size over the whole experiment, in all thermal treatments, by trimming to ~500 eggs. Assuming an equal sex ratio, ~250 eggs would have produced females, in each of the discrete generations in our experiment. Except for the thermal treatment, all other conditions (densities, nutrition) provided to the populations were standardized and optimal (i.e. not stressful), and thus we expect that the female effective population size (Nef) may well have approached 250 under these conditions. For example, Buri [86] measured Ne empirically in his small *Drosophila* populations and found the ratio Ne/Nc~ 0.56-0.71. We come to Nef~ 140-178 by using the same ratio for our experimental populations of females (Nf~ 250). The effective population size can be also estimated using the neutral Wright-Fisher model in terms of the empirical variance [87]. The results are very low in FA group, Ne= (97, 73) for the fluctuating-cold and

fluctuating-warm thermal regimes respectively. The estimate is very strict, but Nunney [88] already concluded that special circumstances are required for Ne/Nc<<0.5, such as selection. We then calculated the expected genetic drift according to the neutral Wright-Fisher model [46] using the strict Nef estimates. We confirm that our experiment lays well within the diffusive regime and far from fixation (Fig. A2a). We verified by simulations and diffusive calculations that the genetic drift can be safely neglected in fluctuating-warm (Fig. A2b; P=0.005) and fluctuating-cold (P=0.035) thermal regimes.

thermal regime	19	25	cold	warm
parental freq.	0.5306±0.0161	0.5306±0.0161	0.5306±0.0161	0.5306±0.0161
final freq.	0.5714±0.0277	0.4800±0.0485	0.5907±0.0301	0.3829±0.0517
freq. difference	0.0409±0.0354	-0.0506±0.0537	0.0601±0.0324	-0.1477±0.0464
# of generations	3	7	3	7
s	0.0566±0.0528	-0.0285±0.0303	0.0849±0.0503	-0.0821±0.0256

Table A1: Selection coefficients (s) inferred for ATB group according to the haploid selection model.

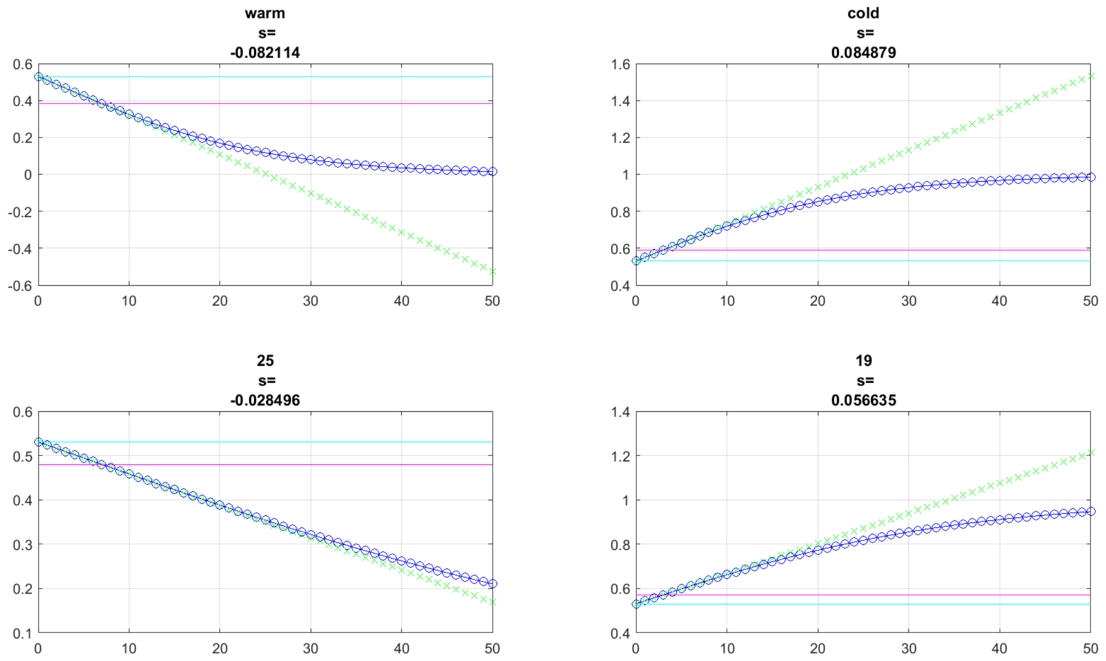

Figure A1 Estimated change in B mtDNA haplogroup frequency within ATB group extrapolated to 50 generations.

Blue – iterative solution of equation (1). Green -linearization with an average slope of θ_i/K. Cyan- initial frequency. Magenta -final frequency.

a)

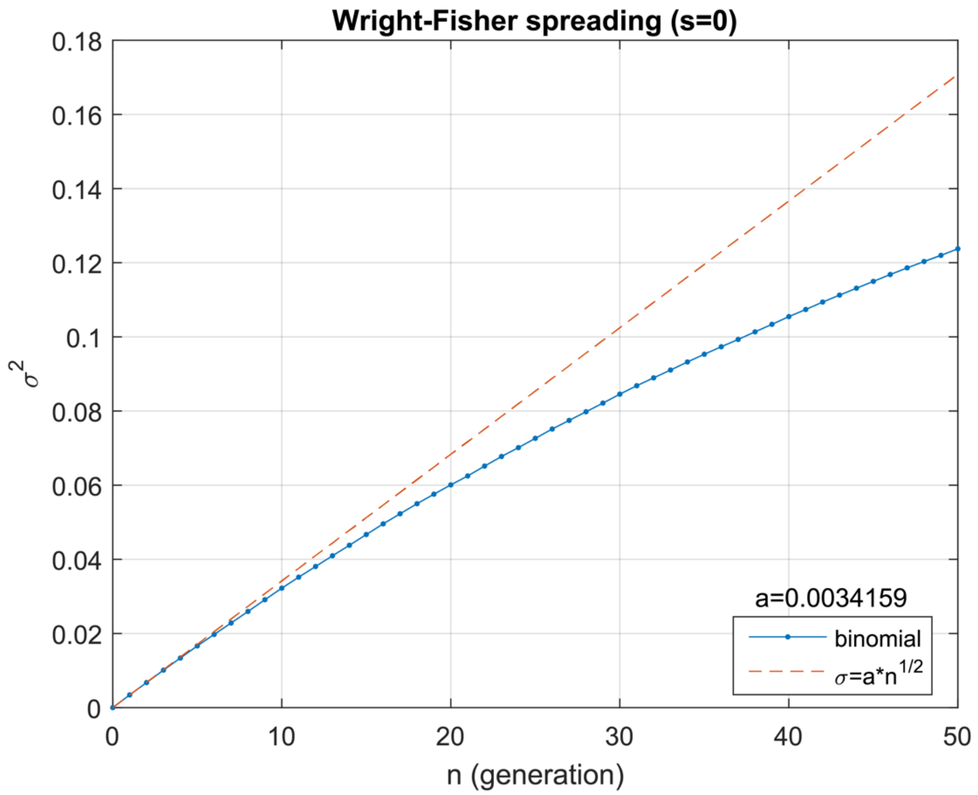
Wright-Fisher spreading (s=0)
0.18
0.16
0.14
0.12
0.1
0.08
0.06
0.04
0.02
0
σ²
0
10
20
30
40
50
n (generation)
a=0.0034159
binomial
σ=a*n^1/2

b)

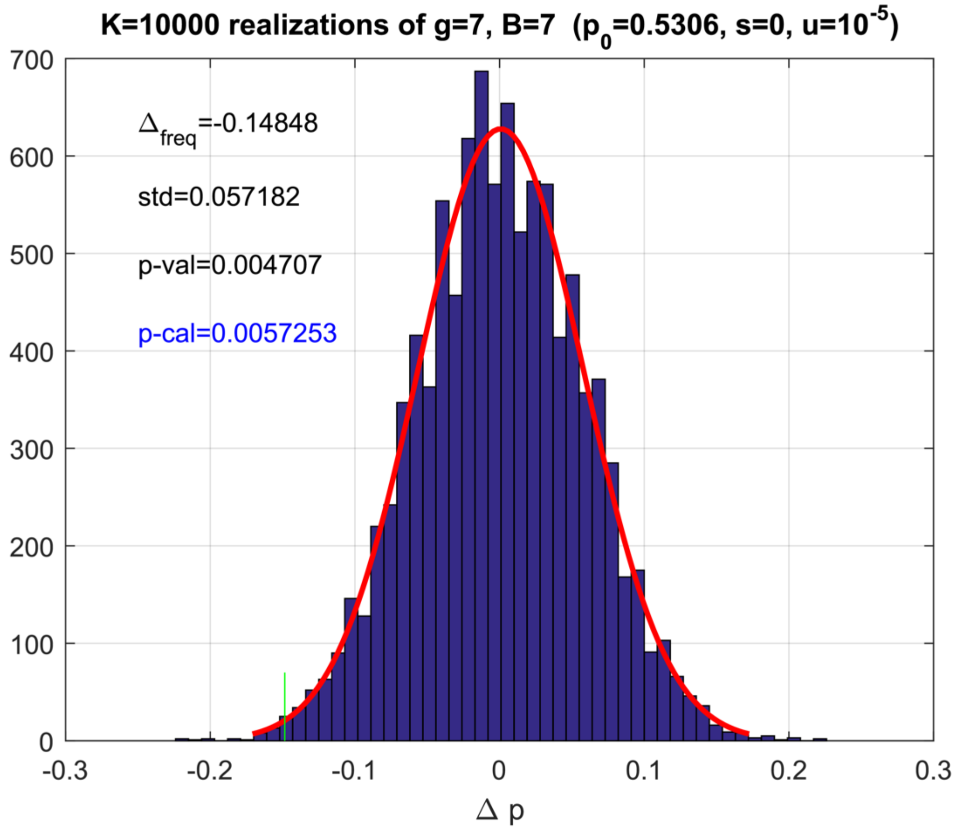
K=10000 realizations of g=7, B=7 (p0=0.5306, s=0, u=10^-5)
700
600
500
400
300
200
100
0
Δfreq=-0.14848
std=0.057182
p-val=0.004707
p-cal=0.0057253
-0.3
-0.2
-0.1
0
0.1
0.2
0.3
Δ p

Figure A2 Examination of genetic drift in fluctuating-warm thermal treatment of our experiment.

We simulated 10,000 realisations of the Wright-Fisher model [46] (50 generations, 7 replicates, 70 constant female effective population size). The simulation shows that in our experiment (7 generations) spreading due to genetic drift is small and lay well within the range of the diffusion approximation **(a)**. The probability of obtaining a frequency shift of $\Delta p = -0.14$ (green mark) in the absence of selection (s=0) is then p-val=0.0047, in good agreement with the diffusive calculation p-cal=0.0057 **(b)**.

References

[86] Buri, P. Gene frequency in small populations of mutant Drosophila. *Evolution* **10**, 367–402 (1956).

[87] Jónás, A., Taus, T., Kosiol, C., Schlotterer, C. & Futschik, A. Estimating the effective population size from temporal allele frequency changes in experimental evolution. *Genetics* **204**, 723-735 (2016).

[88] Nunney, L. The influence of mating system and overlapping generations on effective population size. *Evolution* **47**, 1329-1341 (1993).

Supplementary Table S1: Fly food composition.

**Corn flour based fly food**	
Water (l)	1
Agar (g)	8
Glucose (g)	100
Corn flour (g)	45
Dry yeast (g)	40
Propionic Acid (ml)	4
10% Ethanol solution of Butyl p-Hydroxybenzoate (ml)	3
**Potato starch based fly food**	
Water (l)	1
Agar (g)	7
Dextrose (g)	30
Potato starch (g)	22
Dry yeast (g)	35
Propionic Acid (ml)	5
Nipagen (ml)	12

Supplementary Table S2: List of samples.

In experimental populations ATB1-ATB7, the ancestors had been exposed to antibiotic treatment, while experimental populations UTR1-UTR8 correspond with untreated flies.

Experimental population	Thermal regime	Generation	Sex	Individuals genotyped	A haplogroup	B haplogroup
UTR1	fluctuating warm	7	males	25	22	3
UTR1	fluctuating cold	3	males	26	22	4
UTR1	constant 25°C	7	males	25	11	14
UTR1	constant 19°C	3	males	26	20	6
UTR2	fluctuating warm	7	males	25	5	20
UTR2	fluctuating cold	3	males	25	7	18
UTR2	constant 25°C	7	males	26	15	11
UTR2	constant 19°C	3	males	25	17	8
UTR3	fluctuating warm	7	males	26	8	18
UTR3	fluctuating cold	3	males	28	20	8
UTR3	constant 25°C	7	males	25	8	17
UTR3	constant 19°C	3	males	25	13	12
UTR4	fluctuating warm	7	males	25	0	25
UTR4	fluctuating cold	3	males	26	13	13
UTR4	constant 25°C	7	males	25	7	18
UTR4	constant 19°C	3	males	26	9	17
UTR5	fluctuating warm	7	males	25	8	17
UTR5	fluctuating cold	3	males	25	8	17
UTR5	constant 25°C	7	males	25	12	13
UTR5	constant 19°C	3	males	25	10	15
UTR6	fluctuating warm	7	males	25	13	12
UTR6	fluctuating cold	3	males	25	11	14
UTR6	constant 25°C	7	males	25	2	23
UTR6	constant 19°C	3	males	25	6	19
UTR7	fluctuating warm	7	males	25	10	15
UTR7	fluctuating cold	3	males	25	13	12
UTR7	constant 25°C	7	males	25	3	22
UTR7	constant 19°C	3	males	25	10	15
UTR8	fluctuating warm	7	males	25	7	18
UTR8	fluctuating cold	3	males	26	16	10
UTR8	constant 25°C	7	males	25	6	19
UTR8	constant 19°C	3	males	25	9	16
ATB1	fluctuating warm	7	males	24	19	5
ATB1	fluctuating cold	3	males	26	12	14
ATB1	constant 25°C	7	males	25	11	14
ATB1	constant 19°C	3	males	25	9	16
ATB2	fluctuating warm	7	males	25	12	13
ATB2	fluctuating cold	3	males	25	14	11
ATB2	constant 25°C	7	males	25	19	6
ATB2	constant 19°C	3	males	25	17	8
ATB3	fluctuating warm	7	males	25	7	18
ATB3	fluctuating cold	3	males	26	18	8
ATB3	constant 25°C	7	males	25	16	9
ATB3	constant 19°C	3	males	25	12	13
ATB4	fluctuating warm	7	males	25	12	13
ATB4	fluctuating cold	3	males	26	14	12
ATB4	constant 25°C	7	males	25	16	9

ATB4	constant 19°C	3	males	25	10	15
ATB5	fluctuating warm	7	males	25	18	7
ATB5	fluctuating cold	3	males	25	13	12
ATB5	constant 25°C	7	males	25	9	16
ATB5	constant 19°C	3	males	25	11	14
ATB6	fluctuating warm	7	males	25	10	15
ATB6	fluctuating cold	3	males	25	13	12
ATB6	constant 25°C	7	males	25	12	13
ATB6	constant 19°C	3	males	26	9	17
ATB7	fluctuating warm	7	males	25	8	17
ATB7	fluctuating cold	3	males	25	14	11
ATB7	constant 25°C	7	males	25	9	16
ATB7	constant 19°C	3	males	25	15	10
UTR1	fluctuating warm	7	females	25	21	4
UTR1	fluctuating cold	3	females	26	22	4
UTR1	constant 25°C	7	females	25	14	11
UTR1	constant 19°C	3	females	25	18	7
UTR2	fluctuating warm	7	females	25	11	14
UTR2	fluctuating cold	3	females	25	7	18
UTR2	constant 25°C	7	females	25	16	9
UTR2	constant 19°C	3	females	25	18	7
UTR3	fluctuating warm	7	females	25	6	19
UTR3	fluctuating cold	3	females	25	17	8
UTR3	constant 25°C	7	females	25	13	12
UTR3	constant 19°C	3	females	25	11	14
UTR4	fluctuating warm	7	females	25	4	21
UTR4	fluctuating cold	3	females	25	8	17
UTR4	constant 25°C	7	females	25	11	14
UTR4	constant 19°C	3	females	25	5	20
UTR5	fluctuating warm	7	females	25	9	16
UTR5	fluctuating cold	3	females	25	8	17
UTR5	constant 25°C	7	females	25	15	10
UTR5	constant 19°C	3	females	26	9	17
UTR6	fluctuating warm	7	females	25	10	15
UTR6	fluctuating cold	3	females	25	10	15
UTR6	constant 25°C	7	females	25	5	20
UTR6	constant 19°C	3	females	25	6	19
UTR7	fluctuating warm	7	females	25	16	9
UTR7	fluctuating cold	3	females	25	13	12
UTR7	constant 25°C	7	females	25	10	15
UTR7	constant 19°C	3	females	26	15	11
UTR8	fluctuating warm	7	females	25	5	20
UTR8	fluctuating cold	3	females	26	17	9
UTR8	constant 25°C	7	females	25	9	16
UTR8	constant 19°C	3	females	25	5	20
ATB1	fluctuating warm	7	females	25	18	7
ATB1	fluctuating cold	3	females	26	10	16
ATB1	constant 25°C	7	females	25	12	13
ATB1	constant 19°C	3	females	25	10	15
ATB2	fluctuating warm	7	females	25	17	8
ATB2	fluctuating cold	3	females	25	11	14
ATB2	constant 25°C	7	females	25	12	13
ATB2	constant 19°C	3	females	25	12	13
ATB3	fluctuating warm	7	females	25	12	13
ATB3	fluctuating cold	3	females	25	14	11
ATB3	constant 25°C	7	females	25	19	6
ATB3	constant 19°C	3	females	25	13	12
ATB4	fluctuating warm	7	females	25	19	6

ATB4	fluctuating cold	3	females	25	10	15
ATB4	constant 25°C	7	females	25	15	10
ATB4	constant 19°C	3	females	25	10	15
ATB5	fluctuating warm	7	females	25	18	7
ATB5	fluctuating cold	3	females	25	11	14
ATB5	constant 25°C	7	females	25	14	11
ATB5	constant 19°C	3	females	25	7	18
ATB6	fluctuating warm	7	females	25	15	10
ATB6	fluctuating cold	3	females	25	7	18
ATB6	constant 25°C	7	females	25	8	17
ATB6	constant 19°C	3	females	25	11	14
ATB7	fluctuating warm	7	females	25	9	16
ATB7	fluctuating cold	3	females	25	9	16
ATB7	constant 25°C	7	females	25	11	14
ATB7	constant 19°C	3	females	25	12	13
UTR1	constant 25°C	0	females	31	19	12
UTR2	constant 25°C	0	females	40	18	22
UTR3	constant 25°C	0	females	46	25	21
UTR4	constant 25°C	0	females	47	18	29
UTR5	constant 25°C	0	females	49	16	33
UTR6	constant 25°C	0	females	49	18	31
UTR7	constant 25°C	0	females	50	23	27
UTR8	constant 25°C	0	females	46	21	25
ATB1	constant 25°C	0	females	39	20	19
ATB2	constant 25°C	0	females	49	26	23
ATB3	constant 25°C	0	females	49	22	27
ATB4	constant 25°C	0	females	46	19	27
ATB5	constant 25°C	0	females	48	24	24
ATB6	constant 25°C	0	females	50	23	27
ATB7	constant 25°C	0	females	50	21	29
UTR1	constant 25°C	0	males	43	22	21
UTR2	constant 25°C	0	males	39	15	24
UTR3	constant 25°C	0	males	46	24	22
UTR4	constant 25°C	0	males	46	11	35
UTR5	constant 25°C	0	males	44	15	29
UTR6	constant 25°C	0	males	47	13	34
UTR7	constant 25°C	0	males	48	23	25
UTR8	constant 25°C	0	males	47	16	31
ATB1	constant 25°C	0	males	33	19	14
ATB2	constant 25°C	0	males	43	21	22
ATB3	constant 25°C	0	males	42	19	23
ATB4	constant 25°C	0	males	44	28	16
ATB5	constant 25°C	0	males	47	21	26
ATB6	constant 25°C	0	males	48	22	26
ATB7	constant 25°C	0	males	46	20	26
Townswille (H)	natural	wild	females	20	13	7
Melbourne (C)	natural	wild	females	20	5	15
total				4410	2030	2380

Supplementary Table S3: Foundation dates of experimental populations.

Foundation date marks the date at which virgin flies were combined in a bottle as outlined in Admixture Step 2 (Fig. 1) to form the starting generation. In Admixture Step 1, we allowed their parents to lay eggs for about 1 day, and transferred them to a new bottle. This process was repeated across nine days. We call the process by which we transfer the flies to a new bottle a "tip". Virgin flies of each sex were sourced from several tips, in order to ensure we had an adequate supply of flies to initiate the experimental populations. We show the dates of maternal ovipositioning and virgin collection in a separate column (date). Number means the number of virgin flies sourced from the tip.

Experimental population	Foundation	Virgins type		Virgins source								
code	date	sex	bottle	tip	date	number	tip	date	number	tip	date	number
UTR1	27.3.2013	males	WC	1	5.-16.3.2013	25						
		females	WC	1	5.-16.3.2013	17	2	7.-19.3.2013	8			
		males	CW	1	5.-16.3.2013	25						
		females	CW	1	5.-16.3.2013	25						
UTR2	27.3.2013	males	WC	2	7.-19.3.2013	25						
		females	WC	2	7.-19.3.2013	14	1	5.-18.3.2013	11			
		males	CW	2	7.-19.3.2013	25						
		females	CW	2	7.-19.3.2013	14	1	5.-18.3.2013	11			
UTR3	5.4.2013	females	WC	3	11.-21.3.2013	25						
		males	WC	3	11.-21.3.2013	25						
		females	CW	3	11.-21.3.2013	25						
		males	CW	3	11.-21.3.2013	25						
UTR4	5.4.2013	females	WC	3	11.-21.3.2013	25						
		males	WC	3	11.-21.3.2013	25						
		females	CW	3	11.-21.3.2013	25						
		males	CW	3	11.-21.3.2013	25						
UTR5	5.4.2013	females	WC	3	11.-21.3.2013	25						
		males	WC	3	11.-21.3.2013	25						
		females	CW	3	11.-21.3.2013	25						
		males	CW	3	11.-21.3.2013	25						
UTR6	5.4.2013	females	WC	4	12.-22.3.2013	15	5	12.-23.3.2013	10			
		males	WC	4	12.-22.3.2013	23	5	12.-22.3.2013	2			
		females	CW	4	12.-22.3.2013	15	5	12.-23.3.2013	10			
		males	CW	4	12.-22.3.2013	23	5	12.-22.3.2013	2			
UTR7	5.4.2013	females	WC	6	13.-23.3.2013	18	6	13.-22.3.2013	7			
		males	WC	6	13.-22.3.2013	14	6	13.-23.3.2013	11			
		females	CW	6	13.-23.3.2013	18	6	13.-22.3.2013	7			
		males	CW	6	13.-22.3.2013	14	6	13.-23.3.2013	11			
UTR8	5.4.2013	females	WC	4	12.-22.3.2013	12	3	11.-21.3.2013	13			
		males	WC	6	13.-23.3.2013	25						
		females	CW	4	12.-22.3.2013	12	3	11.-21.3.2013	13			
		males	CW	6	13.-23.3.2013	25						
ATB1	27.3.2013	females	WC	1	5.-16.3.2013	7	2	7.-19.3.2013	4	1	5.-18.3.2013	14
		males	WC	1	5.-16.3.2013	13	2	7.-19.3.2013	12			
		females	CW	1	5.-16.3.2013	7	2	7.-19.3.2013	4	1	5.-18.3.2013	14
		males	CW	1	5.-16.3.2013	13	2	7.-19.3.2013	12			
ATB2	4.4.2013	females	WC	3	11.-21.3.2013	25						
		males	WC	3	11.-21.3.2013	25						
		females	CW	3	11.-21.3.2013	25						
		males	CW	3	11.-21.3.2013	25						
ATB3	4.4.2013	females	WC	3	11.-21.3.2013	25						
		males	WC	3	11.-21.3.2013	25						
		females	CW	3	11.-21.3.2013	25						
		males	CW	3	11.-21.3.2013	25						
ATB4	4.4.2013	females	WC	3	11.-21.3.2013	25						
		males	WC	3	11.-21.3.2013	25						
		females	CW	3	11.-21.3.2013	25						
		males	CW	3	11.-21.3.2013	25						
ATB5	4.4.2013	females	WC	4	12.-22.3.2013	25						
		males	WC	4	12.-22.3.2013	25						
		females	CW	4	12.-22.3.2013	25						
		males	CW	4	12.-22.3.2013	25						
ATB6	4.4.2013	females	WC	6	13.-23.3.2013	25						
		males	WC	6	13.-23.3.2013	25						
		females	CW	6	13.-23.3.2013	25						
		males	CW	6	13.-23.3.2013	25						
ATB7	4.4.2013	females	WC	6	13.-23.3.2013	25						
		males	WC	6	13.-23.3.2013	25						
		females	CW	6	13.-23.3.2013	25						
		males	CW	6	13.-23.3.2013	25						

Supplementary Table S4: Propagation of experimental populations in dates.

Foundation date corresponds with Supplementary Table S3. We imposed the four different thermal regimes on multiple generations starting by eggs laid by the starting generation. Within each generation, the flies of each experimental bottle were transferred to new bottles, and the column "tip number" reflects the "tip" that was used to propagate the next generation per experimental population. Although the tip we used to initiate each experimental population varied in Generation 0, in subsequent generations, we propagated experimental populations mostly from the first tip.

Experimental population	Foundation	Conditions	Generation0		Generation1		Generation2		Generation3		Generation4		Generation5		Generation6		Generation7	
code	date		tip	time/date	tip	time/date	tip	time/date	tip/sample(+)	time/date	tip	time/date	tip	time/date	tip	time/date	sample(+)	time/date
UTR1	27.3.2013	constant 25°C	3	12:00/5.4.2013	2	12:00/22.4.2013	1	14:00/5.5.2013	1	17:00/20.5.2013	1	14:00/4.6.2013	1	18:00/17.6.2013	1	20:00/5.7.2013	+	23:00/18.7.2013
		constant 19°C	6	19:00/8.4.2013	2	15:30/2.5.2013	1	18:00/1.6.2013	+	11:00/3.7.2013								
		fluctuating warm	5	13:00/8.4.2013	1	13:00/19.4.2013	1	10:30/30.4.2013	1	14:00/13.5.2013	1	20:00/26.5.2013	2	13:00/12.6.2013	1	19:00/29.6.2013	+	12:00/18.7.2013
		fluctuating cold	7	11:00/9.4.2013	2	14:30/6.5.2013	1	17:00/6.6.2013	+	01:00/8.7.2013								
UTR2	27.3.2013	constant 25°C	3	12:00/5.4.2013	2	12:00/22.4.2013	1	14:00/5.5.2013	1	17:00/20.5.2013	1	14:00/4.6.2013	1	18:00/17.6.2013	1	20:00/5.7.2013	+	23:00/18.7.2013
		constant 19°C	6	19:00/8.4.2013	2	15:30/2.5.2013	1	18:00/1.6.2013	+	11:00/3.7.2013								
		fluctuating warm	5	13:00/8.4.2013	1	13:00/19.4.2013	1	10:30/30.4.2013	1	14:00/13.5.2013	1	20:00/26.5.2013	2	13:00/12.6.2013	1	19:00/29.6.2013	+	12:00/18.7.2013
		fluctuating cold	7	11:00/9.4.2013	2	14:30/6.5.2013	1	17:00/6.6.2013	+	01:00/8.7.2013								
UTR3	5.4.2013	constant 25°C	2	17:30/7.4.2013	2	12:00/22.4.2013	1	14:00/5.5.2013	1	17:00/20.5.2013	1	14:00/4.6.2013	1	18:00/17.6.2013	1	21:00/5.7.2013	+	23:00/18.7.2013
		constant 19°C	5	19:00/8.4.2013	2	15:30/2.5.2013	1	18:00/1.6.2013	+	11:00/3.7.2013								
		fluctuating warm	4	13:00/8.4.2013	1	13:00/19.4.2013	1	10:30/30.4.2013	1	14:00/13.5.2013	1	20:00/26.5.2013	2	13:00/12.6.2013	1	20:00/29.6.2013	+	12:00/18.7.2013
		fluctuating cold	3	0:30/8.4.2013	2	14:00/2.5.2013	1	18:00/1.6.2013	+	14:00/3.7.2013								
UTR4	5.4.2013	constant 25°C	2	17:30/7.4.2013	2	12:00/22.4.2013	1	14:00/5.5.2013	1	17:00/20.5.2013	1	14:00/4.6.2013	1	18:00/17.6.2013	1	21:00/5.7.2013	+	23:00/18.7.2013
		constant 19°C	5	19:00/8.4.2013	2	15:30/2.5.2013	1	18:00/1.6.2013	+	11:00/3.7.2013								
		fluctuating warm	4	13:00/8.4.2013	1	13:00/19.4.2013	1	10:30/30.4.2013	1	14:00/13.5.2013	1	20:00/26.5.2013	2	13:00/12.6.2013	1	20:00/29.6.2013	+	12:00/18.7.2013
		fluctuating cold	3	0:30/8.4.2013	2	14:00/2.5.2013	1	18:00/1.6.2013	+	14:00/3.7.2013								
UTR5	5.4.2013	constant 25°C	2	17:30/7.4.2013	2	12:00/22.4.2013	1	14:00/5.5.2013	1	17:00/20.5.2013	1	14:00/4.6.2013	1	18:00/17.6.2013	1	21:00/5.7.2013	+	23:00/18.7.2013
		constant 19°C	5	19:00/8.4.2013	2	15:30/2.5.2013	1	18:00/1.6.2013	+	11:00/3.7.2013								
		fluctuating warm	4	13:00/8.4.2013	1	13:00/19.4.2013	1	10:30/30.4.2013	1	14:00/13.5.2013	1	20:00/26.5.2013	2	13:00/12.6.2013	1	20:00/29.6.2013	+	12:00/18.7.2013
		fluctuating cold	3	0:30/8.4.2013	2	14:00/2.5.2013	1	18:00/1.6.2013	+	14:00/3.7.2013								
UTR6	5.4.2013	constant 25°C	2	17:30/7.4.2013	2	12:00/22.4.2013	1	14:00/5.5.2013	1	17:00/20.5.2013	1	14:00/4.6.2013	1	18:00/17.6.2013	1	21:00/5.7.2013	+	23:00/18.7.2013
		constant 19°C	5	19:00/8.4.2013	2	15:30/2.5.2013	1	18:00/1.6.2013	+	11:00/3.7.2013								
		fluctuating warm	4	13:00/8.4.2013	1	13:00/19.4.2013	1	10:30/30.4.2013	1	14:00/13.5.2013	1	20:00/26.5.2013	2	13:00/12.6.2013	1	20:00/29.6.2013	+	12:00/18.7.2013
		fluctuating cold	3	0:30/8.4.2013	2	14:00/2.5.2013	1	18:00/1.6.2013	+	14:00/3.7.2013								
UTR7	5.4.2013	constant 25°C	2	17:30/7.4.2013	2	12:00/22.4.2013	1	14:00/5.5.2013	1	17:00/20.5.2013	1	14:00/4.6.2013	1	18:00/17.6.2013	1	21:00/5.7.2013	+	23:00/18.7.2013
		constant 19°C	5	19:00/8.4.2013	2	15:30/2.5.2013	1	18:00/1.6.2013	+	11:00/3.7.2013								
		fluctuating warm	4	13:00/8.4.2013	1	13:00/19.4.2013	1	10:30/30.4.2013	1	14:00/13.5.2013	1	20:00/26.5.2013	2	13:00/12.6.2013	1	20:00/29.6.2013	+	12:00/18.7.2013
		fluctuating cold	3	0:30/8.4.2013	2	14:00/2.5.2013	1	18:00/1.6.2013	+	14:00/3.7.2013								
UTR8	5.4.2013	constant 25°C	2	17:30/7.4.2013	2	12:00/22.4.2013	1	14:00/5.5.2013	1	17:00/20.5.2013	1	14:00/4.6.2013	1	18:00/17.6.2013	1	21:00/5.7.2013	+	23:00/18.7.2013
		constant 19°C	5	19:00/8.4.2013	2	15:30/2.5.2013	1	18:00/1.6.2013	+	11:00/3.7.2013								
		fluctuating warm	4	13:00/8.4.2013	1	13:00/19.4.2013	1	10:30/30.4.2013	1	14:00/13.5.2013	1	20:00/26.5.2013	2	13:00/12.6.2013	1	20:00/29.6.2013	+	12:00/18.7.2013
		fluctuating cold	3	0:30/8.4.2013	2	14:00/2.5.2013	1	18:00/1.6.2013	+	14:00/3.7.2013								
ATB1	27.3.2013	constant 25°C	3	12:00/5.4.2013	2	12:00/22.4.2013	1	14:00/5.5.2013	1	17:00/20.5.2013	1	14:00/4.6.2013	1	18:00/17.6.2013	1	20:00/5.7.2013	+	23:00/18.7.2013
		constant 19°C	6	19:00/8.4.2013	2	15:00/2.5.2013	1	18:00/1.6.2013	+	11:00/3.7.2013								
		fluctuating warm	5	13:00/8.4.2013	1	13:00/19.4.2013	1	10:30/30.4.2013	1	14:00/13.5.2013	1	20:00/26.5.2013	2	13:00/12.6.2013	1	19:00/29.6.2013	+	16:00/18.7.2013
		fluctuating cold	7	11:00/9.4.2013	2	14:30/6.5.2013	1	17:00/6.6.2013	+	01:00/8.7.2013								
ATB2	4.4.2013	constant 25°C	2	17:30/7.4.2013	2	12:00/22.4.2013	1	14:00/5.5.2013	1	17:00/20.5.2013	1	14:00/4.6.2013	1	18:00/17.6.2013	1	21:00/5.7.2013	+	23:00/18.7.2013
		constant 19°C	5	19:00/8.4.2013	2	15:30/2.5.2013	1	18:00/1.6.2013	+	11:00/3.7.2013								
		fluctuating warm	4	13:00/8.4.2013	1	13:00/19.4.2013	1	10:30/30.4.2013	1	14:00/13.5.2013	1	20:00/26.5.2013	2	13:00/12.6.2013	1	20:00/29.6.2013	+	16:00/18.7.2013
		fluctuating cold	3	0:30/8.4.2013	2	14:00/2.5.2013	1	18:00/1.6.2013	+	14:00/3.7.2013								
ATB3	4.4.2013	constant 25°C	2	17:30/7.4.2013	2	12:00/22.4.2013	1	14:00/5.5.2013	1	17:00/20.5.2013	1	14:00/4.6.2013	1	18:00/17.6.2013	1	21:00/5.7.2013	+	23:00/18.7.2013
		constant 19°C	5	19:00/8.4.2013	2	15:30/2.5.2013	1	18:00/1.6.2013	+	11:00/3.7.2013								
		fluctuating warm	4	13:00/8.4.2013	1	13:00/19.4.2013	1	10:30/30.4.2013	1	14:00/13.5.2013	1	20:00/26.5.2013	2	13:00/12.6.2013	1	20:00/29.6.2013	+	16:00/18.7.2013
		fluctuating cold	3	0:30/8.4.2013	2	14:00/2.5.2013	1	18:00/1.6.2013	+	14:00/3.7.2013								
ATB4	4.4.2013	constant 25°C	2	17:30/7.4.2013	2	12:00/22.4.2013	1	14:00/5.5.2013	1	17:00/20.5.2013	1	14:00/4.6.2013	1	18:00/17.6.2013	1	21:00/5.7.2013	+	23:00/18.7.2013
		constant 19°C	5	19:00/8.4.2013	2	15:30/2.5.2013	1	18:00/1.6.2013	+	11:00/3.7.2013								
		fluctuating warm	4	13:00/8.4.2013	1	13:00/19.4.2013	1	10:30/30.4.2013	1	14:00/13.5.2013	1	20:00/26.5.2013	2	13:00/12.6.2013	1	20:00/29.6.2013	+	16:00/18.7.2013
		fluctuating cold	3	0:30/8.4.2013	2	14:00/2.5.2013	1	18:00/1.6.2013	+	14:00/3.7.2013								
ATB5	4.4.2013	constant 25°C	2	17:30/7.4.2013	2	12:00/22.4.2013	1	14:00/5.5.2013	1	17:00/20.5.2013	1	14:00/4.6.2013	1	18:00/17.6.2013	1	21:00/5.7.2013	+	23:00/18.7.2013
		constant 19°C	5	19:00/8.4.2013	2	15:30/2.5.2013	1	18:00/1.6.2013	+	11:00/3.7.2013								
		fluctuating warm	4	13:00/8.4.2013	1	13:00/19.4.2013	1	10:30/30.4.2013	1	14:00/13.5.2013	1	20:00/26.5.2013	2	13:00/12.6.2013	1	20:00/29.6.2013	+	16:00/18.7.2013
		fluctuating cold	3	0:30/8.4.2013	2	14:00/2.5.2013	1	18:00/1.6.2013	+	14:00/3.7.2013								
ATB6	4.4.2013	constant 25°C	2	17:30/7.4.2013	2	12:00/22.4.2013	1	14:00/5.5.2013	1	17:00/20.5.2013	1	14:00/4.6.2013	1	18:00/17.6.2013	1	21:00/5.7.2013	+	23:00/18.7.2013
		constant 19°C	5	19:00/8.4.2013	2	15:30/2.5.2013	1	18:00/1.6.2013	+	11:00/3.7.2013								
		fluctuating warm	4	13:00/8.4.2013	1	13:00/19.4.2013	1	10:30/30.4.2013	1	14:00/13.5.2013	1	20:00/26.5.2013	2	13:00/12.6.2013	1	20:00/29.6.2013	+	16:00/18.7.2013
		fluctuating cold	3	0:30/8.4.2013	2	14:00/2.5.2013	1	18:00/1.6.2013	+	14:00/3.7.2013								
ATB7	4.4.2013	constant 25°C	2	17:30/7.4.2013	2	12:00/22.4.2013	1	14:00/5.5.2013	1	17:00/20.5.2013	1	14:00/4.6.2013	1	18:00/17.6.2013	1	21:00/5.7.2013	+	23:00/18.7.2013
		constant 19°C	5	19:00/8.4.2013	2	15:30/2.5.2013	1	18:00/1.6.2013	+	11:00/3.7.2013								
		fluctuating warm	4	13:00/8.4.2013	1	13:00/19.4.2013	1	10:30/30.4.2013	1	14:00/13.5.2013	1	20:00/26.5.2013	2	13:00/12.6.2013	1	20:00/29.6.2013	+	16:00/18.7.2013
		fluctuating cold	3	0:30/8.4.2013	2	14:00/2.5.2013	1	18:00/1.6.2013	+	14:00/3.7.2013								

Supplementary Table S5: Selected SNPs characteristics for mtDNA haplogroups.

Red colour marks the site recognized by *HinfI* restriction enzyme used for haplogroups recognition by Boussy et al. in 1998 [47] in the A haplogroup that appears more frequent in warmer areas of Australia [24].

SNP code (position)	SNP type	Gene	Aminoacid	Position in triplet	A haplogroup	B haplogroup
1154	C/T	ND2	Asparagine	3	C	T
2661	C/T	COX1	Proline	3	C	T
3583	C/T	COX2	Alanine	3	T	C
4247	C/T	ATP6	Glycine	3	C	T
5396	C/T	COX3	Leucine	1	C	T
6299	A/C	trnE	-	-	C	A
6980	A/G	ND5	Tyrosine	3	G	A
7862	A/G	ND5	Phenylalanine	3	G	A
8867	A/G	ND4	Leucine	1	A	G
8973	C/T	ND4	Leucine	3	C	T
10217	C/T	ND6	Leucine	1	C	T
10673	C/T	CYTB	Leucine	1	T	C
12123	C/T	ND1	Methionine	3	C	T
12336	A/C	ND1	Glycine	3	C	A
